# In-Field, In Situ, and In Vivo 3-Dimensional Elemental Mapping for Plant Tissue and Soil Analysis Using Laser-Induced Breakdown Spectroscopy

**DOI:** 10.3390/s16101764

**Published:** 2016-10-22

**Authors:** Chunjiang Zhao, Daming Dong, Xiaofan Du, Wengang Zheng

**Affiliations:** National Engineering Research Center for Information Technology in Agriculture, Beijing Academy of Agriculture and Forestry Sciences, Beijing 100097, China; zhaocj@nercita.org.cn (C.Z.); duxf@nercita.org.cn (X.D.); zhengwg@nercita.org.cn (W.Z.)

**Keywords:** in-field, in situ, in vivo, elemental mapping, laser-induced breakdown spectroscopy

## Abstract

Sensing and mapping element distributions in plant tissues and its growth environment has great significance for understanding the uptake, transport, and accumulation of nutrients and harmful elements in plants, as well as for understanding interactions between plants and the environment. In this study, we developed a 3-dimensional elemental mapping system based on laser-induced breakdown spectroscopy that can be deployed in- field to directly measure the distribution of multiple elements in living plants as well as in the soil. Mapping is performed by a fast scanning laser, which ablates a micro volume of a sample to form a plasma. The presence and concentration of specific elements are calculated using the atomic, ionic, and molecular spectral characteristics of the plasma emission spectra. Furthermore, we mapped the pesticide residues in maize leaves after spraying to demonstrate the capacity of this method for trace elemental mapping. We also used the system to quantitatively detect the element concentrations in soil, which can be used to further understand the element transport between plants and soil. We demonstrate that this method has great potential for elemental mapping in plant tissues and soil with the advantages of 3-dimensional and multi-elemental mapping, in situ and in vivo measurement, flexible use, and low cost.

## 1. Introduction

Sensing and imaging elemental concentrations in biological tissues has great importance for understanding uptake, transport, and accumulation of both nutritious and harmful elements in plants as well as cell absorption and formation processes [1]. These elements include macro nutrient elements, which are the major elements that compose tissues; micro nutrient elements; and toxic elements. In the past few decades, scientists have continuously researched novel elemental imaging and mapping methods for biological tissues [1,2,3,4,5,6]. Rapid, in situ, high-resolution, and sensitive imaging are the main trends being explored among elemental imaging methods.

Currently, three types of elemental imaging and mapping methods are used for biological tissues: X-ray microscopy-based methods [2,7,8], mass spectroscopy-based methods [2,9,10,11,12] and fluorescent probes [13,14]. For the X-ray microscopy methods, the commonly used methods are scanning or transmission electron microscopy with energy-dispersive X-ray analysis (SEM-EDX or TEM-EDX) and proton/particle-induced X-ray emission (PIXE) [2,7]. The former requires a vacuum sampling system, but the latter can only analyze elements lighter than Na. Upon development and establishment of synchrotron radiation (SR) systems, certain researchers have used X-ray fluorescence spectroscopy based on synchrotron radiation (SRXRF) and X-ray absorption spectroscopy based on synchrotron radiation (SRXAS) to image elements in plant tissues through the SR beam-line [8,15,16,17]. The SR beam-line provides a detection limit of ppm (Parts-per-million) and a space resolution near 100 nm, but one difficultly is access to a SR beam-line [18]. Secondary ionization mass spectrometry (SIMS) is the most-used mass spectroscopy method for elemental imaging in biological tissues [2,9,19]. Nano-SIMS was developed in recent years which provides much higher spatial resolution than common SIMS [2]. However, SIMS requires high vacuum conditions, and a quantitative analysis is difficult due to strong matrix effects. Certain scholars have used laser ablation inductively coupled plasma mass spectrometry (LA-ICP-MS) and laser micro-dissection inductively coupled plasma mass spectrometry (LMD-ICP-MS) for elemental compositions in solid surfaces [10,11,20,21,22,23], but these methods provide only a few applications for plant tissue. One potential basis for the limited application is the dehydration effects on the samples during the laser ablation processes, which render the plant sample unable to maintain moisture and an intact structure.

Notably, the above methods depend on complex and expensive experimental instruments and systems and can only be performed in a laboratory. In certain plant research fields, scientists hope to map elements in plant tissues in-field and in vivo, which may lead to a better understanding of the transport and accumulation laws governing nutrient and toxic elements in plants. However, the aforementioned methods cannot provide such imaging and mapping. Consider imaging pesticide distribution in plant tissues as an example, which we will discuss below. Mapping and quantitative observation of pesticide residues in plants is important for studies on absorption, transport, and accumulation of pesticides in plants. However, only a few studies used matrix-assisted laser desorption/ionization (MALDI) mass spectrometry to image the pesticide on leaf surfaces [24,25,26], which requires a sample of the leaves first and then a spray of the matrix solution. Therefore, it is impossible to produce in situ measurements.

Therefore, an in situ and in vivo micro-imaging and mapping method for both high-concentration and trace elements in plant tissues is needed which can be used in-field. Laser-induced breakdown spectroscopy (LIBS) is a fast-developing spectroscopy method that analyzes elemental compositions and concentrations through inducing the measured objects into plasma using a high-energy laser, and then collects the emission spectra [27,28]. LIBS has been used to qualitatively and quantitatively measure solids, liquids, and gases based on atomic, ionic, and molecular spectral characteristics [28,29,30,31]. LIBS has also been used for fast mapping and imaging elemental distributions on solid surfaces [27,32,33,34,35,36,37,38,39]. Compared with other imaging and mapping methods, LIBS is advantageous due to its simple structure, flexibility in use, and low cost, and it can be used in-field, is removable, and can even be used as a handheld device [28,40]. In this study, we developed a removable elemental mapping system based on micro-LIBS, LipsImag. We demonstrated quantitative elemental mapping through LipsImag in plant tissues using maize leaves after a pesticide spray as an example, and we also used LipsImag to detect the element concentrations in soil. To the best of our knowledge, this is the first method and system for in situ and in vivo 3-dimensional elemental mapping of biological tissues that is removable and can be used in-field.

## 2. Experimental Methods

### 2.1. LipsImag

**Figure 1:**
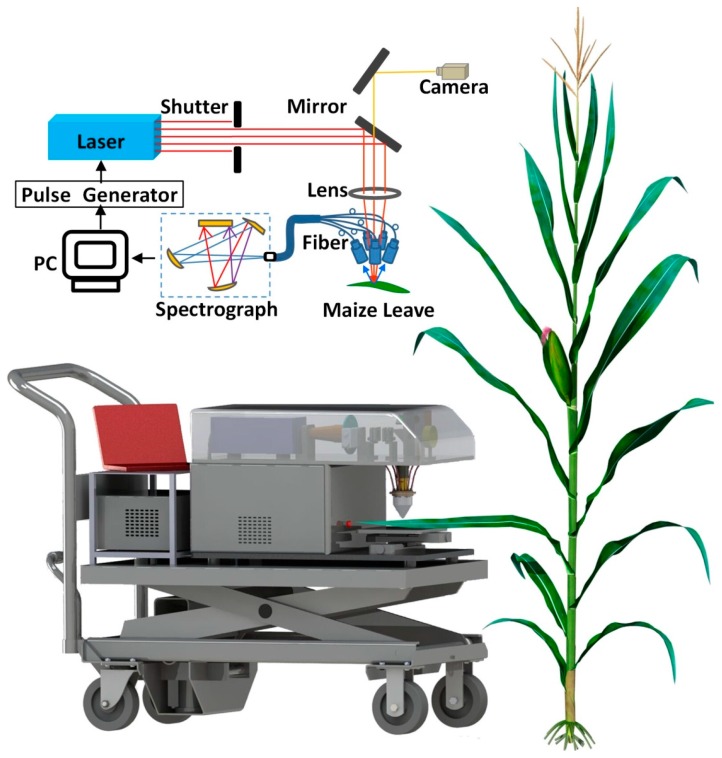
Schematic of the LipsImag apparatus and its application for in situ and in vivo elemental mapping of maize.

The principles of LipsImag are as follows: (1) a laser beam from a laser generator emits to the sample, focuses on its surface and induces a micro area into plasma status; (2) the emitted plasma spectrum is detected by a spectrometer; (3) the presence and concentrations of some objective elements are calculated using the spectral characteristics; (4) the focusing area of the laser beam moves to the next point using a 3-dimensional movement platform and repeat the above steps until all of the points in the scanning area of the first plane are measured; (5) the height of the movement platform is lowered to the next plane and the above steps are repeated until all the planes are scanned or the sample is penetrated. The schematic of the LipsImag apparatus is shown in Figure 1. In our study, we used a CFR200 laser (Quantel Ltd., Les Ulis, France) to output a 1064 nm laser beam with a maximum energy of 200 mJ. A sevenbands spectrometer, HR2000+ (Ocean Optics, Dunedin, FL, USA), was used to collect the emitted plasma spectra with a resolution of 0.2 nm and a spectral band of 200–850 nm. We tested the spectrometer in-field, and validated its SNR (signal-to-noise ratio) as approximately 170.94. To perform 3-dimensional scanning, a precision 3-dimensional stage, LX-8375 (Felles Photonic Instruments, Shanghai, China), was used in LipsImag with an accuracy of 7 μm. To observe the surface of the sample and to adjust the height of the movement platform, a Grasshopper3 (LUSTER LightTech, Beijing, China) camera was used which was coaxial with the laser focusing system. We also designed a removable stand to equip all of the components above, as well as a computer to control the laser shot, spectral collection, and platform movement, and to calculate the mapping results automatically. The control and calculation software of LipsImag was developed in the VS2010 platform.

### 2.2. Samples

**Figure 2:**
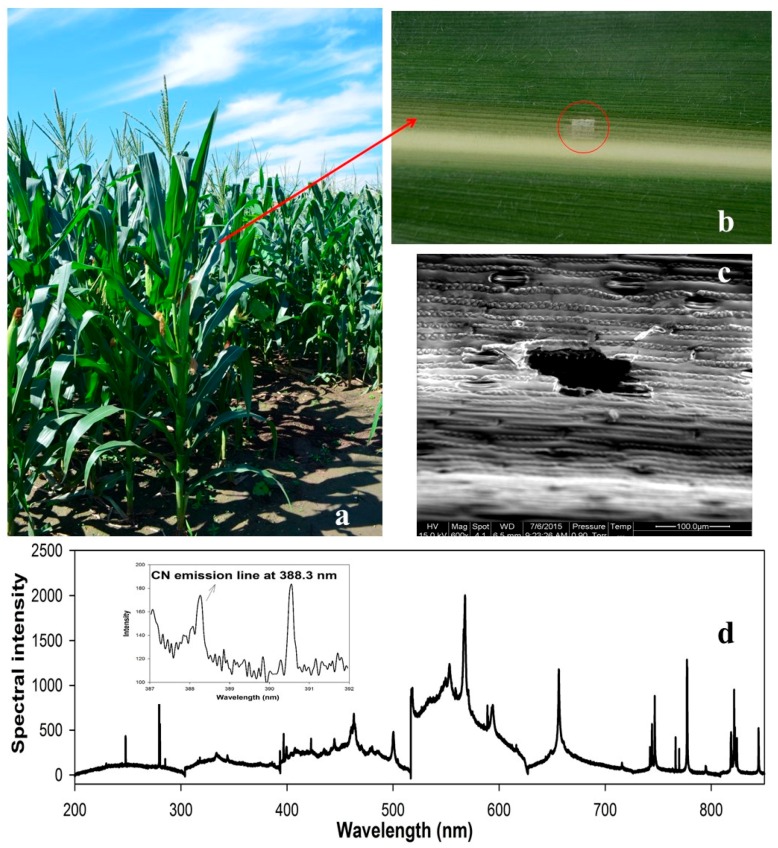
The photographs in a real application of LipsImag for an in-field measurement. (**a**) Maize plants for the elemental mapping experiment; (**b**) Photograph of a maize leaf after a 20 × 20 scanning by LipsImag; (**c**) The microstructure of a single shot on a maize leaf by LipsImag; (**d**) A plasma emission spectra from the scanning that is shown in (**b**).

As shown in Figure 2, the maize plants used in our study were grown naturally in the experimental field of the Beijing Academy of Agriculture and Forestry (Beijing, China). The living plants were directly used in the experiment. We also used small holly (*Ilex chinensis Sims*) leaves to demonstrate the ability of LipsImag for nutrient elemental mapping, which was also grown in the Beijing Academy of Agriculture and Forestry. Chlorpyrifos (C_9_H_11_Cl_3_NO_3_PS) emulsifiable concentrate (EC) that we used in the experiment was produced by Dow AgroSciences Ltd. (Indianapolis, IN, USA) with a concentration of 40.7%. To prepare chlorpyrifos with different concentrations, we diluted the pesticide various times using water. The commonly used concentration in our study was 100 mL chlorpyrifos EC (40.7%) diluted by 50 L of water and sprayed in a 1 mu (666.7 m^2^) maize field. We also used some lower concentrations to study the detection sensitivity of LipsImag. The spray was carried out using a 5372 sprayer (Worth Ltd., Shanghai, China) following the guidelines of the Chinese government. The soil samples were obtained from farmland in the Haidian District (Beijing, China). To avoid splashing, each soil sample was pressed into a tablet (diameter of 13 mm, 1 mm thick) under the pressure of 10 T with a tablet machine (FW-4A, Xin Tian Guang Ltd., Tianjin, China). In this study, 60 groups of samples were prepared. The real values of element contents were tested by the Beijing Research Centre for Agriculture and Testing.

### 2.3. Experiments

The field experiment was carried out by moving LipsImag outdoor, stretching the living maize leaves onto the platform, fixing them with double-faced adhesive tape and then scanning. The holly leaves cannot be stretched due to a distance problem and thus were cut and then placed on the platform to complete the scanning. For the calibration experiment, a 20 µL pesticide sample which was diluted several different times, from 400 to 40,000, were dropped onto the surface of the maize leaf in an area of about 1 cm^2^. In this way, we obtained the samples with pesticide residues of 0–30 µg/cm^2^. Then, we used LipsImag to measure the intensities of the spectral characteristics and establish a calibration model. In this study, we used a 90 mJ laser energy, a 75 µm laser spot size and a 200–850 nm spectral band in most cases.

### 2.4. Spectral Processing and Mapping Methods

The spectral processing algorithms were embedded in the software of LipsImag. The spectra were processed by the following steps: (1) the background line was subtracted from the plasma emission spectra [28]; (2) the spectrum was smoothed [28]; (3) each spectra band was area-normalized in a 20 nm window to decrease the influence of the laser jitter [40]; (4) the area of each spectral characteristics peak was calculated as a variable for calibration [28]. After scanning, the presence and concentrations of the specific elements of each point in the 3-dimensional scanning array had been calculated, then, 2D surface images, 3D images, and 2D cross-sectional images that show the distribution of the elements in plant tissues were drawn.

## 3. Results and Discussion

### 3.1. In Situ and In Vivo 3-Dimensional Elemental Mapping of Plant Leaves

LipsImag consists of an LIBS module, a 3-dimensional movement platform, an observation camera, and a removable stand, as shown in Figure 1. The measurements are generated by the LIBS module as follows: An Nd:YAG laser outputs a 1064 nm laser pulse onto the micro area of the measured object. The micro area will then be induced into a plasma, the plasma signal will be collected by a fiber and dispersed by gratings, an emission spectrum will be acquired, and, finally, the element concentrations will be calculated. The observation camera is used to observe the sample surface micro-structure and is coaxial with the laser. Figure 1 shows how LipsImag measures maize leaves in-field. We stretched a living leaf onto the 3-dimensional movement platform and fixed it with double-faced adhesive tape. Next, the distance between the sample and laser was adjusted using the observation camera. The spot size and scanning areas can be selected using the LipsImag operating software. During the measurement, the first micro point in the first plane was hit by the laser, and the spectrum was measured; next, each point in the scanning area was hit and measured one-by-one through automatically adjusting the 3-dimensional movement platform. When scanning for the first plane was completed, the height of the 3-dimensional platform was automatically adjusted, and LipsImag began scanning the next plane. Thus, a collection of spectra that correspond to a 3-dimensional micro area can be acquired, and a 3-dimensional elemental distribution can be imaged. Certain leaves and stalks must be cut and then placed onto the platform if they are unable to be stretched. Notably, the LipsImag is much simpler than mass spectroscopy-based and X-ray microscopy-based imaging and mapping systems.

The photographs that we collected using LipsImag for field measurements of maize leaves are shown in Figure 2. In this experiment, we used a 90 mJ laser energy, and the spot size was 75 µm. Figure 2c shows the surface morphology of a single point measurement, which was visualized using scanning electron microscopy. The laser ablation area comprises an ellipse with a longer diameter of approximately 120 µm, which may be generated by the leaf tissue structure. The ablation depth was approximately 12 µm, which was measured using atomic force microscopy and scanning electron microscopy. Figure 2b shows a photograph collected after scanning 20 × 20 points and 4 planes. In this measurement, the distance between each spot was 200 µm to avoid overlap and an interrelationship. Thus, the scanning area for a single plane was 4 × 4 mm. When scanning for the first plane was completed, the 3-dimensional platform moved 12 µm up to scan the next plane. Figure 2b shows that the laser ablation produced only a small amount of damage to the maize leaves, which may not influence its growth.

**Figure 3:**
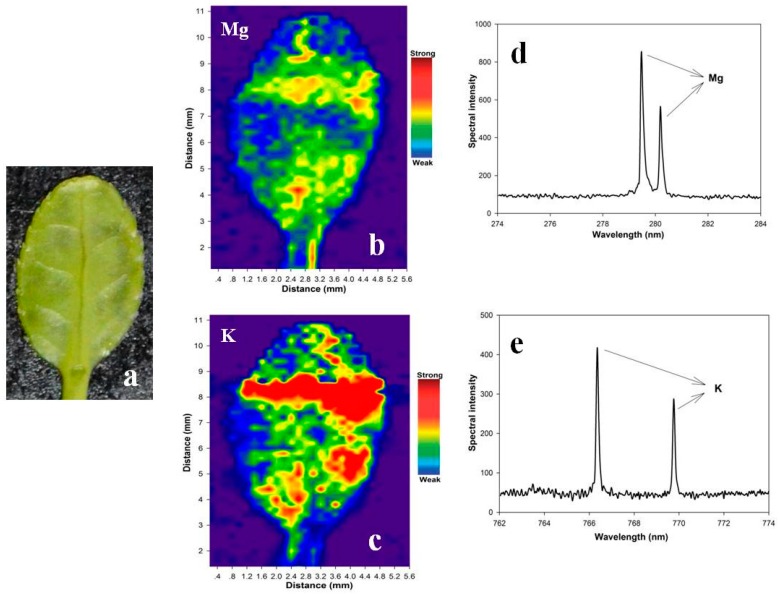
The mapping results of holly (Ilex chinensis Sims) leaf based on the spectral intensities of specific elements using LipsImag. (**a**) The visible image of the leaf for the mapping experiment; (**b**) and (**c**) shows the distributions of the spectral intensities of Mg and K, respectively; (**d**,**e**) illustrate the spectral characteristics of Mg and K that were used for mapping.

Figure 2d shows a plasma emission spectrum that corresponds to Figure 2b. The elemental concentrations can be calculated based on the atomic and ionic spectral characteristics [28]. Moreover, certain spectral characteristics belong to the molecular fragments, e.g., CN, which can be used to directly analyze the concentrations of certain specific molecules [41]. LipsImag can be used to image the distributions for the presence and concentrations of certain nutritional elements, e.g., Mg, Ca, and Na, and for toxic elements at trace concentrations (such as P in pesticide). Figure 3 shows the mapping results for the small holly leaf measurements, in which the distributions of the spectral intensities for Mg, Ca, and Na are clear. Notably, these are the pre-calibration results, and the spectral intensities are used instead for elemental concentrations. In practice, a calibration is performed before field use and is based on samples with known concentrations that are used to construct a quantitative model using spectral characteristics and elemental concentrations. Next, the calibration model was installed in the LipsImag software, through which the elemental concentration distributions can be imaged in real time. Notably, LipsImag operates at a much higher spatial resolution, e.g., 20 µm, but the sensitivity and detection limit are lower due to the laser ablation mass decrease. We will use LipsImag with leaves to detect pesticide residues that are considered trace elements; thus, we used a 200 µm resolution throughout the analysis.

### 3.2. Chlorpyrifos Residue Spectral Characteristics in Plants and Quantitative Analyses Thereof

As discussed previously, measuring pesticide distributions in plant tissue is important for plant and environmental research. The previous mapping method, i.e., MALDI, can only detect pesticides on the leaf surface, not inside of a leaf [26]. More importantly, these methods are difficult to use for mapping in-field and in vivo due to their complex structure. Most pesticides contain specific elements (e.g., P and S), which thus provides an opportunity for mapping the elements’ concentrations in plant tissues using LipsImag [30,42]. In this study, we used chlorpyrifos, which is a common pesticide in China, as an example and attempted to collect 3-dimentional images of its distribution in plant leaves.

**Figure 4:**
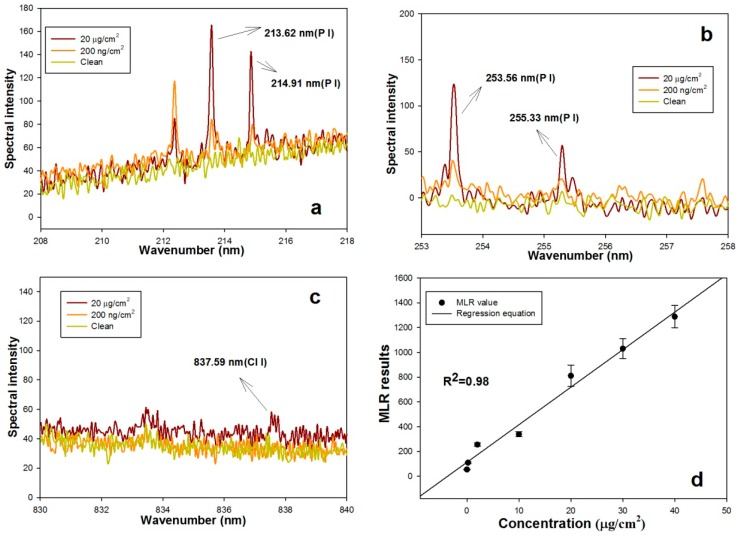
The laser-induced plasma spectral characteristics of chlorpyrifos in a leaf. (**a**) The spectral characteristics of the element P at 213.62 nm and 214.91 nm; (**b**) The spectral characteristics of the element P at 253.56 nm and 255.33 nm; (**c**) The spectral characteristics of the element Cl at 827.59 nm; (**d**) The multi-variable linear regression model for chlorpyrifos measurement based on the spectral characteristics of P and Cl.

Chlorpyrifos (C_9_H_11_Cl_3_NO_3_PS) contains several elements, including P, S, H, Cl, O, N, and C. H, C, O, and N also exist in air and plant tissue. Thus, we attempted to determine the presence of and calculate the concentrations of chlorpyrifos using the spectral characteristics of P, S, and Cl. Figure 4 shows the plasma emission spectra from LipsImag for a leaf with 20 µg/cm^2^ and 200 ng/cm^2^ chlorpyrifos residue and a clean leaf. The spectral characteristics of P at 213.62 nm, 214.91 nm, 253.56 nm, and 255.33 nm are shown in Figure 4a,b, and the spectral characteristics of Cl at 827.59 nm are shown in Figure 4c. The spectral characteristics for P at the four wavelengths were clear in the leaf samples with pesticide but were absent in the clean leaf, and the intensities increased with higher concentration samples. However, the Cl spectral line was much weaker such that it was only observed in samples with 20 µg/cm^2^ chlorpyrifos. We attempted to discern the spectral characteristics of the S atom and ion, but the characteristics were not found. Spectral characteristics of P and S in the ultraviolet band are much stronger than their visible band characteristics [43,44,45]. However, an ultraviolet spectrometer requires a vacuum environment; thus, it is difficult to use in-field. Furthermore, to measure the pesticide more precisely, we constructed a multi-variable regression model using samples with a known pesticide concentration and the spectral characteristics of P and Cl (Figure 4d).

### 3.3. Two-Dimensional Mapping of Chlorpyrifos on a Leaf Surface

**Figure 5:**
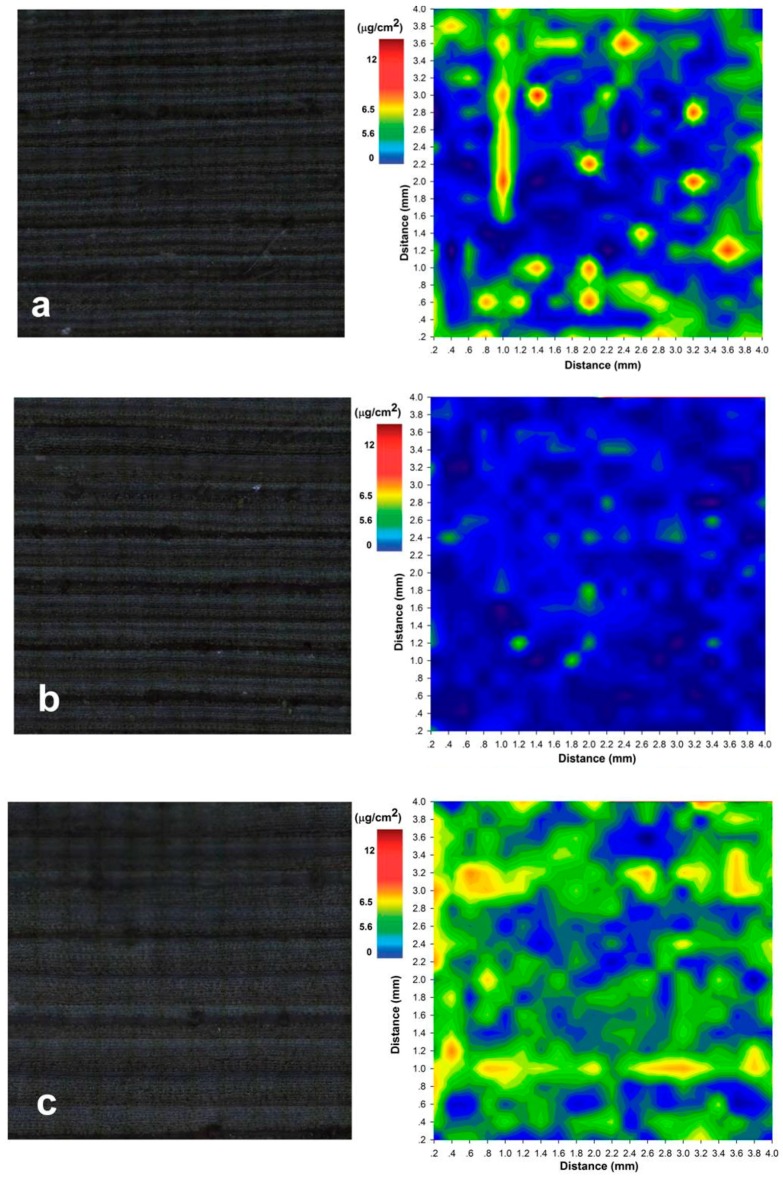
The 2D mapping results of chlorpyrifos on a leaf surface using LipsImag. (**a**) The mapping result of pesticide on a leaf that used 100 mL chlorpyrifos EC (40.7%) diluted by 50 L water and was subsequently sprayed in a 1 mu (666.7 m^2^) maize field; (**b**) The mapping result of a lower concentration of pesticide that used 10 mL chlorpyrifos; (**c**) The mapping result of the leaf 10 h after spray application that used 100 mL chlorpyrifos EC. The left image of (**a**–**c**) shows the visible image observed by microscopy, while the right image is the scanning results that show the distribution of the pesticide.

We used LipsImag to image the pesticide distribution on a leaf surface after the calibration model was constructed and installed. Figure 5a shows the mapping results for a random maize leaf after a spray application, which included 100 mL chlorpyrifos EC (40.7%) diluted with 50 L water and spayed in a 1 mu (666.7 m^2^) maize field. This dose is common and is a concentration that prevents aphids and leaf mites in China. In this experiment, we stretched the leaves onto the platform 20 min after the spray and collected the measurements. The scanning area was 20 × 20 points with a 200 µm distance between each point. The distributions of certain chlorpyrifos droplets are clear in Figure 5a, and certain vertical bands may have been caused by the droplets falling under gravity. Figure 5b also includes mapping results but with a lower pesticide dose at 10 mL chlorpyrifos EC (40.7%), which was also diluted with 50 L water. Therefore, the pesticide residue in Figure 5b is at much lower levels than in Figure 5a. We also used LipsImag to study the processes of chlorpyrifos spread and transport on a leaf surface through mapping a leaf 10 h after spraying (100 mL chlorpyrifos EC), as shown in Figure 5c. Clearly, the droplet concentrations are weaker, but the area is greater. Furthermore, certain horizontal bands appear, which may be caused by water transport in the leaf vein.

### 3.4. Three-Dimensional Mapping of Chlorpyrifos in Leaves

**Figure 6:**
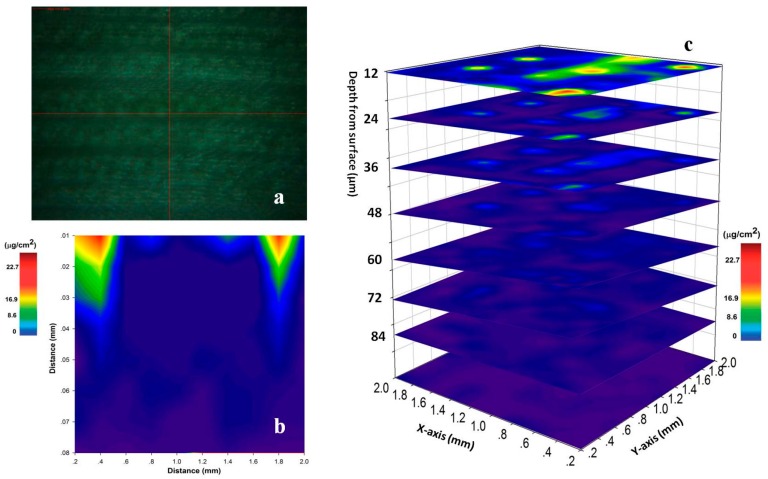
The 3-dimensional mapping of pesticide residues in a maize leaf 10 h after spray. (**a**) is the visible image of the scanning area observed by microscopy; (**b**) is a cross-sectional image that shows the pesticide distribution in a lateral plane; (**c**) shows the 3-dimensional image of the pesticide by scanning one plane after another, with a thickness of 12 µm for each plane.

LipsImag can provide 3-dimensional elemental mapping for laser ablation depth [27,35,36,37,45]. As discussed above, the laser ablation depth of the maize leaf is approximately 12 µm with a 90 mJ laser and a 75 µm spot size. The experiment was performed 10 h after spraying (100 mL chlorpyrifos EC). We imaged the chlorpyrifos distribution in a leaf through scanning one plane after another with a 12 µm thickness in each plane. A thicker part near the middle of the leaf was used to avoid penetration. Figure 6c shows mapping results for pesticide residues that were thicker than in Figure 5a, which may be due to the easy enrichment of the droplets in the middle of the leaf. The pesticide concentration clearly deceased with depth, and almost no pesticide was detected from the fifth plane. To deeply understand the processes of pesticide permeation in plants, we used a horizontal band in Figure 6b and drew a cross-sectional image to show the pesticide distribution in a lateral plane (Figure 6c). Clearly, the chlorpyrifos gradually decreased and disappeared at a 50 µm depth. It is known that the laser ablation will change the physical properties of the leaf and influence the measurements among two layers. However, the influence was not too much in our experiment when we used 12 µm as a step size in depth. To demonstrate the above issue, we reproduced Figure 6b in the same conditions but with an opposite measuring direction (from the bottom to the top). The distribution of pesticide shows a similar shape in that that the pesticide only appears in the first three layers.

### 3.5. Measurement of the Element Concentrations in Soil Using LipsImag

**Figure 7:**
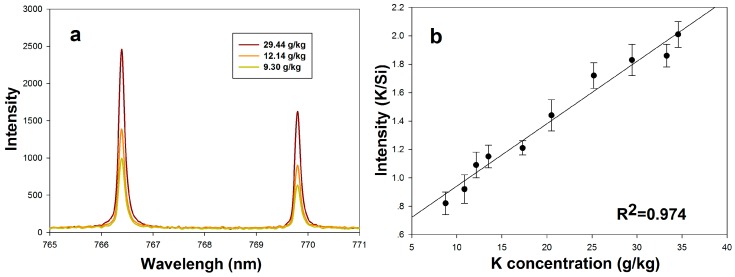
Determination of K in soil using LipsImag. (**a**) shows the spectral characteristics of K in soil; (**b**) is the calibration model for K concentration measurement.

To provide an ability to further understand the element transportation processes among soil and plant tissues, we also used the LipsImag system to detect element presence and concentrations in soil. The soil was sampled and squashed into a tablet to avoid splashing. We studied the spectral characteristics of K, Mg, Na, and Ca. and the calibration models were built. The concentration of Si in soil is stable, so we used the spectral line at 288.16 nm of Si as an internal standard and established calibration models to reduce the influence of laser jitter and soil moisture. Figure 7 shows the spectral characteristics and the calibration model of K in soil.

## 4. Conclusions

This study offers a new elemental mapping method to botanists besides X-ray microscopy-based methods, mass spectroscopy based-methods, and fluorescent probes. Because it can be moved to the field and can measure living plants directly, the distribution of nutrients and harmful elements in plant tissues as well as the interactions between plants and the environment can be studied naturally without sample cultivation, collection, and pre-treatment. The laser will ablate the sample during measurement, but the ablation area is small and will not influence the plant growth; thus, it can be considered a micro-destructive or even non-destructive measurement tool. The above experiments and results demonstrate that LipsImag can provide 3-dimensional mapping for elemental distributions in living plant tissues. MALDI-MS was also used for pesticide imaging in plant leaves with a spatial resolution of 300 μm × 300 μm [26], which is lower than LipsImag. MALDI-MS images the pesticide distribution based on molecular structure while LIBS is based on atomic concentration. More importantly, LipsImag has much simpler optical structures compared to MALDI-MS and thus can be moved to the field to measure the elemental distribution of living plants. LIBS is comparable to LA-ICP-MS because both are based on laser ablation. A main restriction for LA-ICP-MS applications in plant research is dehydration effects; thus, related studies have focused on experimental conditions that maintain sample moisture and structure [2,22,23]. However, this problem is avoided by using LipsImag to directly measure living plants under growth conditions. Moreover, LipsImag includes certain other advantages, such as a simultaneous analysis of multiple elements and low cost, compared to other methods.

Notably, this is the first study where LIBS was applied to in situ and in vivo elemental mapping of plants; thus, further studies are necessary. In this study, we used a 200 µm spatial resolution, which is lower than previously used methods, e.g., SRXRF and SIMS [8,9,16]. In fact, LipsImag can operate at a much higher resolution (10–50 µm) to image smaller objects, e.g., cells and microorganisms. We used a 200 µm resolution due to the trace concentrations of the pesticide in the plant leaves, which are difficult to detect using a lower energy laser and smaller spot size. We will attempt to use a higher resolution in future studies for mapping nutrient elements in plant tissues. Another issue that must be considered is the detection limit of the LIBS method, which can reach ppm levels for solid analyses under conventional conditions [28]. Using a vacuum environment, buffered gases and a multi-pulse laser can enhance the LIBS detection sensitivity but lead to difficulties with in-field and in vivo measurements [27,28,30]. During the LIBS measurement process, the plasma spectrum contains spectral characteristics of atom, iron, and molecule fragments, but we only used the atomic spectral line in this study. For certain specific measurements, the spectral characteristics of molecular fragments can be observed and used to directly image the molecular distributions [41,46,47,48]. Theoretically, LIBS can measure nearly all elements, but it is a problem for elements that exist in air, e.g., C, H, and O. One potential way to avoid the influence of air is by using a vacuum environment or buffered gases [49]. Other methods may also be used to measure these elements, such as establishing particular models based on chemometrics, which must be considered under specific conditions and for specific applications [28,48].

## References

[1] Zhao F.J., Moore K.L., Lombi E., Zhu Y.G. (2014). Imaging element distribution and speciation in plant cells. Trends Plant Sci..

[2] Wu B., Becker J.S. (2012). Imaging techniques for elements and element species in plant science. Metallomics.

[3] Waldron K.J., Rutherford J.C., Ford D., Robinson N.J. (2009). Metalloproteins and metal sensing. Nature.

[4] Hare D.J., New E.J., de Jonge M.D., McColl G. (2015). Imaging metals in biology: Balancing sensitivity, selectivity and spatial resolution. Chem. Soc. Rev..

[5] Van de Plas R., Yang J., Spraggins J., Caprioli R.M. (2015). Image fusion of mass spectrometry and microscopy: A multimodality paradigm for molecular tissue mapping. Nat. Methods.

[6] Eliceiri K.W., Berthold M.R., Goldberg I.G., Ibáñez L., Manjunath B.S., Martone M.E., Murphy R.F., Peng H., Plant A.L., Roysam B. (2012). Biological imaging software tools. Nat. Methods.

[7] Ingall E.D., Diaz J.M., Longo A.F., Oakes M., Finney L., Vogt S., Lai B., Yager P.L., Twining B.S., Brandes J.A. (2013). Role of biogenic silica in the removal of iron from the Antarctic seas. Nat. Commun..

[8] Isaure M.P., Fraysse A., Devès G., le Lay P., Fayard B., Susini J., Bourguignon J., Ortega R. (2006). Micro-chemical imaging of cesium distribution in Arabidopsis thaliana plant and its interaction with potassium and essential trace elements. Biochimie.

[9] Angelo M., Bendall S.C., Finck R., Hale M.B., Hitzman C., Borowsky A.D., Levenson R.M., Lowe J.B., Liu S.D., Zhao S. (2014). Multiplexed ion beam imaging of human breast tumors. Nat. Med..

[10] Becker J.S., Becker J.S. (2008). Quantitative images of metals in plant tissues measured by laser ablation inductively coupled plasma mass spectrometry. Spectrochim. Acta B At. Spectrosc..

[11] Becker J.S., Zoriy M., Wu B., Matuschb A., Becker J.S. (2008). Imaging of essential and toxic elements in biological tissues by LA-ICP-MS. J. Anal. At. Spectrom..

[12] Huang R., Zhang B., Zou D., Hang W., He J., Huang B. (2011). Elemental imaging via laser ionization orthogonal time-of-flight mass spectrometry. Anal. Chem..

[13] Chan J., Dodani S.C., Chang C.J. (2012). Reaction-based small-molecule fluorescent probes for chemoselective bioimaging. Nat. Chem..

[14] Swanson S.J., Choi W.G., Chanoca A., Gilroy S. (2011). In vivo imaging of Ca^2+^, pH, and reactive oxygen species using fluorescent probes in plants. Annu. Rev. Plant Biol..

[15] Karunakaran C., Lahlali R., Zhu N., Webb A.M., Schmidt M., Fransishyn K., Belev G., Wysokinski T., Olson J., Cooper D.M. (2015). Factors influencing real time internal structural visualization and dynamic process monitoring in plants using synchrotron-based phase contrast X-ray imaging. Sci. Rep..

[16] Kim H.K., Lee S.J. (2010). Synchrotron X-ray imaging for nondestructive monitoring of sap flow dynamics through xylem vessel elements in rice leaves. New Phytol..

[17] Terzano R., Alfeld M., Janssens K., Vekemans B., Schoonjans T., Vincze L., Tomasi N., Pinton R., Cesco S. (2013). Spatially resolved (semi)quantitative determination of iron (Fe) in plants by means of synchrotron micro X-ray fluorescence. Anal. Bioanal. Chem..

[18] Wernet P., Kunnus K., Josefsson I., Rajkovic I., Quevedo W., Beye M., Schreck S., Grübel S., Scholz M., Nordlund D. (2015). Orbital-specific mapping of the ligand exchange dynamics of Fe(CO)_5_ in solution. Nature.

[19] Chaurand P. (2012). Imaging mass spectrometry of thin tissue sections: A decade of collective efforts. J. Proteom..

[20] Da Silva M.A., Arruda M.A. (2013). Laser ablation (imaging) for mapping and determining Se and S in sunflower leaves. Metallomics.

[21] Hare D., Austin C., Doble P. (2012). Quantification strategies for elemental imaging of biological samples using laser ablation-inductively coupled plasma-mass spectrometry. Analyst.

[22] Wu B., Chen Y., Becker J.S. (2009). Study of essential element accumulation in the leaves of a Cu-tolerant plant Elsholtzia splendens after Cu treatment by imaging laser ablation inductively coupled plasma mass spectrometry (LA-ICP-MS). Anal. Chim. Acta.

[23] Wu B., Zoriy M., Chen Y., Becker J.S. (2009). Imaging of nutrient elements in the leaves of Elsholtzia splendens by laser ablation inductively coupled plasma mass spectrometry (LA-ICP-MS). Talanta.

[24] Anderson D.M., Carolan V.A., Crosland S., Sharples K.R., Clench M.R. (2009). Examination of the distribution of nicosulfuron in sunflower plants by matrix-assisted laser desorption/ionisation mass spectrometry imaging. Rapid Commun. Mass Spectrom..

[25] Anderson D.M., Carolan V.A., Crosland S., Sharples K.R., Clench M.R. (2010). Examination of the translocation of sulfonylurea herbicides in sunflower plants by matrix-assisted laser desorption/ionisation mass spectrometry imaging. Rapid Commun. Mass Spectrom..

[26] Annangudi S.P., Myung K., Avila Adame C., Gilbert J.R. (2015). MALDI-MS Imaging Analysis of Fungicide Residue Distributions on Wheat Leaf Surfaces. Environ. Sci. Technol..

[27] Noll R. (2012). Laser-Induced Breakdown Spectroscopy: Fundamentals and Applications.

[28] CREMERS D.A. (2013). Handbook of Laser-Induced Breakdown Spectroscopy.

[29] Bousquet B., Sirven J.B., Canioni L. (2007). Towards quantitative laser-induced breakdown spectroscopy analysis of soil samples. Spectrochim. Acta B At. Spectrosc..

[30] Du X., Dong D., Zhao X., Jiao L., Han P., Lang Y. (2015). Detection of pesticide residues on fruit surfaces using laser induced breakdown spectroscopy. RSC Adv..

[31] Meslin P.Y., Gasnault O., Forni O., Schröder S., Cousin A., Berger G., Clegg S.M., Lasue J., Maurice S., Sautter V. (2013). Soil diversity and hydration as observed by ChemCam at Gale crater, Mars. Science.

[32] Chirinos J.R., Oropeza D.D., Gonzalez J.J., Hou H., Morey M., Zorbaa V., Russo E.R. (2014). Simultaneous 3-dimensional elemental imaging with LIBS and LA-ICP-MS. J. Anal. At. Spectrom..

[33] Kaiser J., Galiová M., Novotný K., Červenka R., Reale L., Novotný J., Liška M., Samek O., Kanický V., Hrdlička A. (2009). Mapping of lead, magnesium and copper accumulation in plant tissues by laser-induced breakdown spectroscopy and laser-ablation inductively coupled plasma mass spectrometry. Spectrochim. Acta B At. Spectrosc..

[34] Sancey L., Motto-Ros V., Busser B., Kotb S., Benoit J.M., Piednoir A., Lux F., Tillement O., Panczer G., Yu J. (2014). Laser spectrometry for multi-elemental imaging of biological tissues. Sci. Rep..

[35] Lucena P., Laserna J.J. (2001). Three-dimensional distribution analysis of platinum, palladium and rhodium in auto catalytic converters using imaging-mode laser-induced breakdown spectrometry. Spectrochim. Acta B At. Spectrosc..

[36] Gimenez Y., Busser B., Trichard F., Kulesza A., Laurent J.M., Zaun V., Lux F., Benoit J.M., Panczer G., Dugourd P. (2016). 3D Imaging of Nanoparticle Distribution in Biological Tissue by Laser-Induced Breakdown Spectroscopy. Sci. Rep..

[37] Papazoglou D.G., Papadakis V., Anglos D. (2004). In situ interferometric depth and topography monitoring in LIBS elemental profiling of multi-layer structures. J. Anal. At. Spectrom..

[38] Wang X., Motto-Ros V., Panczer G., De Ligny D., Yu J., Benoit J.M., Dussossoy J.L., Peuget S. (2013). Mapping of rare earth elements in nuclear waste glass–ceramic using micro laser-induced breakdown spectroscopy. Spectrochim. Acta B At. Spectrosc..

[39] Piñon V., Mateo M.P., Nicolas G. (2013). Laser-induced breakdown spectroscopy for chemical mapping of materials. Appl. Spectrosc. Rev..

[40] Mosier-Boss P.A., Lieberman S.H., Theriault G.A. (2002). Field demonstrations of a direct push FO-LIBS metal sensor. Environ. Sci. Technol..

[41] Fernández-Bravo Á., Delgado T., Lucena P., Laserna J.J. (2013). Vibrational emission analysis of the CN molecules in laser-induced breakdown spectroscopy of organic compounds. Spectrochim. Acta B At. Spectrosc..

[42] Ma F., Dong D. (2014). A Measurement Method on Pesticide Residues of Apple Surface Based on Laser-Induced Breakdown Spectroscopy. Food Anal. Methods.

[43] Kondo H., Hamada N., Wagatsuma K. (2009). Determination of phosphorus in steel by the combined technique of laser induced breakdown spectrometry with laser induced fluorescence spectrometry. Spectrochim. Acta B At. Spectrosc..

[44] Li C.M., Zou Z.M., Yang X.Y., Hao Z.Q., Guo L.B., Li X.Y., Lua Y.F., Zeng X.Y. (2014). Quantitative analysis of phosphorus in steel using laser-induced breakdown spectroscopy in air atmosphere. J. Anal. At. Spectrom..

[45] Milan M., Lucena P., Cabalin L.M. (1998). Depth Profiling of Phosphorus in Photonic-Grade Silicon Using Laser-Induced Breakdown Spectrometry. Appl. Spectrosc..

[46] Delgado T., Vadillo J.M., Laserna J.J. (2013). Laser-induced plasma spectroscopy of organic compounds. Understanding fragmentation processes using ion–photon coincidence measurements. J. Anal. At. Spectrom..

[47] De Lucia F.C., Gottfried J.L. (2010). Characterization of a Series of Nitrogen-Rich Molecules using Laser Induced Breakdown Spectroscopy. Propellants Explos. Pyrotech..

[48] Doucet F.R., Faustino P.J., Sabsabia M., Lyonb R.C. (2008). Quantitative molecular analysis with molecular bands emission using laser-induced breakdown spectroscopy and chemometrics. J. Anal. At. Spectrom..

[49] Harris R.D., Cremers D.A., Ebinger M.H., Bluhm B.K. (2004). Determination of nitrogen in sand using laser-induced breakdown spectroscopy. Appl. Spectrosc..

